# Bioengineering of Microalgae: Recent Advances, Perspectives, and Regulatory Challenges for Industrial Application

**DOI:** 10.3389/fbioe.2020.00914

**Published:** 2020-09-03

**Authors:** Gulshan Kumar, Ajam Shekh, Sunaina Jakhu, Yogesh Sharma, Ritu Kapoor, Tilak Raj Sharma

**Affiliations:** ^1^Agricultural Biotechnology Division, National Agri-Food Biotechnology Institute (NABI), Sahibzada Ajit Singh Nagar, India; ^2^Plant Cell Biotechnology Department, CSIR-Central Food Technological Research Institute (CFTRI), Mysuru, India; ^3^Division of Crop Science, Indian Council of Agricultural Research, New Delhi, India

**Keywords:** microalgae, genetic engineering, *omics*, genome editing, regulatory issues

## Abstract

Microalgae, due to their complex metabolic capacity, are being continuously explored for nutraceuticals, pharmaceuticals, and other industrially important bioactives. However, suboptimal yield and productivity of the bioactive of interest in local and robust wild-type strains are of perennial concerns for their industrial applications. To overcome such limitations, strain improvement through genetic engineering could play a decisive role. Though the advanced tools for genetic engineering have emerged at a greater pace, they still remain underused for microalgae as compared to other microorganisms. Pertaining to this, we reviewed the progress made so far in the development of molecular tools and techniques, and their deployment for microalgae strain improvement through genetic engineering. The recent availability of genome sequences and other omics datasets form diverse microalgae species have remarkable potential to guide strategic momentum in microalgae strain improvement program. This review focuses on the recent and significant improvements in the omics resources, mutant libraries, and high throughput screening methodologies helpful to augment research in the model and non-model microalgae. Authors have also summarized the case studies on genetically engineered microalgae and highlight the opportunities and challenges that are emerging from the current progress in the application of genome-editing to facilitate microalgal strain improvement. Toward the end, the regulatory and biosafety issues in the use of genetically engineered microalgae in commercial applications are described.

## Introduction

The proficient photosynthetic microorganisms including green microalgae, diatoms, and cyanobacteria offer remarkable advantage over the terrestrial plants as a rich source of various biomolecules to be used for food, feed, and fuel applications. In addition to the faster growth rate, higher biomass productivity, and ability to synthesize complex metabolites with minimal resources are some of their key advantages. The wide taxonomic and inherent biochemical diversity among the microalgal species makes them suitable resource of abundant biomolecules with industrial and biomedical importance. Owing to this, microalgae have been continuously exploited for the production of biomolecules such as lipids, proteins, and carbohydrates. Apart from the production of secondary metabolites, microalgae have also been targeted for various applications in nutraceuticals, pharmaceuticals, dietary supplements, and personal care products. Microalgae are also utilized for concomitant CO_2_ sequestration, wastewater treatment, and biomass production for high-volume low-value products (Yadav et al., 2014; Mehar et al., 2019). In the last few years, owing to the high lipid content in microalgae (20–70% of dry cell weight), various start-up companies in the sector of clean energy production have attempted for commercialization of microalgae derived biofuels (Mata et al., 2010; Chisti, 2013). According to a global market research, the market for algal products across various segments is expected to grow at a compound annual growth rate of 4.2% from 2018 to 2025 and will have a total market value of more than 3.4 billion USD (https://www.alliedmarketresearch.com/algae-products-market).

Even though the commercial potential of microalgae along with its market portfolio is well-known, challenges pertaining to its economic feasibility still remain to be addressed. High biomass production along with the desired metabolite(s), cost-efficient dewatering and harvesting of biomass, green and efficient process for product extraction are some of the broad challenges to further improve the microalgal process economics. Among all these, the robust and highly efficient strain with desired characteristics can substantially improve the economics of upstream processing. Though various nutritional-, environmental-, and physiological-alteration-based cultivation have been attempted for improved microalgal productivities, commercial success remains limited (Pierobon et al., 2018). This is mainly due to the fact that these biotechnological amendments in the cultivation processes could not enhance the inherent metabolic capacity of the microalgae to hyperaccumulate the desired metabolite(s). For example, triggering the lipid accumulation in microalgae through nutrient deprivation inevitably lowers the cell division, thereby making it difficult to simultaneously achieve high lipid accumulation and high growth rate, thus decreasing the final lipid productivity (Lenka et al., 2016).

**Figure 1:**
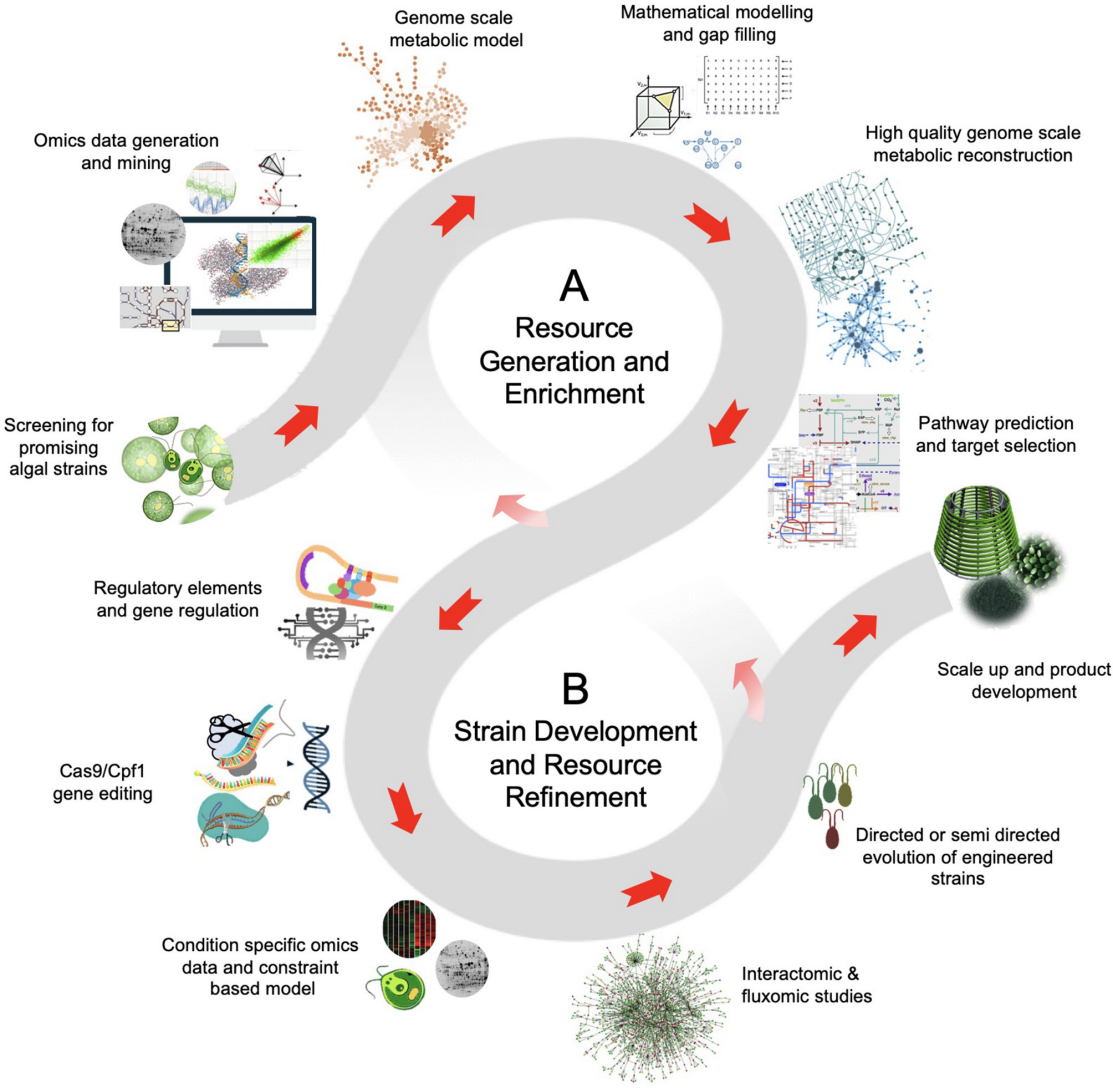
The schematic of data and resource driven strategy for microalgal bioengineering. **(A)** *Resource generation and enrichment:* The high-throughput technologies, intense computation and bioinformatic analysis, and the extensive research interest on microalgae can generate high-quality curated data. The genomic and transcriptomic data of model organisms provides a basic understanding of the biosynthetic pathway. This imperative information is aided by proteomics and metabolomics that offers functional insights for bioproduct discovery in microalgae. Also, the metabolomic data can be implemented to novel microbial isolates with limited genomic and transcriptomic information. **(B)** *Strain development and resource refinement:* The leads from metabolic models and the use of state-of-the-art technologies, such as genome-editing and high-throughput variant selection can be used for microalgae strain development. Often the metabolic flux shifts of the mutants implies an organism's evolution to optimize flux rearrangement. The objective of the flux balance shift can be biomass production or enhanced production of desired product. Moreover, the information obtained from fine-tuned modeling and genomic-editing experiments create resource avenues for further discoveries.

In this context, the genetic engineering of microalgae can help to overcome the inherent limitation of metabolic capacity for higher accumulation of desired biomolecules, thus eventually improving the economic feasibility of the production process. Though the wide taxonomic and genetic diversity among the microalgae offer several opportunities for genetic modifications, the scarcity of genomic resources and genetic tools limits the progress in algal bioengineering. For instance, the information of genome sequence, metabolic pathway maps, and the other genetic resources that are the key to identify target gene(s) is available only for the limited (mostly model) microalgal strains. However, despite the available genome sequence information, the annotation, and the gene functional studies related to the microalgae are still very limited. Since many of the microalgal genome sequences will be studied in near future, the computational biology and the bioinformatics may play an important role in precise genome assembly and its annotation. In addition, the multiomics datasets for microalgae can also be explored to improve the biorefinery capabilities and the quality of the microalgal bioproducts (Fayyaz et al., 2020). Moreover, the functional genetic screening through genome scale mutant libraries and their high-throughput screening may help to make robust strategies for microalgal strain improvement. Therefore, such information is extremely essential for purpose-specific bioengineering of microalgal strains. The typical strategic path from the integration of different datasets to the microalgal strain improvement is illustrated in Figure 1. In the process of genetic-engineering-based strain improvement, the molecular tools for stable transformation, selective screening, and precise gene targeting are extremely important to accomplish the genetic modification. Unlike other microorganisms, such as bacteria, yeast, and fungi, the microalgal bioengineering suffer the lack of efficient genetic tools and techniques.

Considering these shortcomings, in this review, we have thoroughly mapped the information regarding the evolution of genetic modification strategies from the conventional to the emerging genome-editing tools and their implication in microalgae bioengineering. Although the bioengineering of microalgae holds the great potential to improve process economics, the risk assessment, biosafety, and regulatory issues pertaining to the use of genetically engineered microalgae must be considered and are summarized in this review. We attempt to comprehensively describe the resources for microalgae bioengineering, including omics resources, mutant resources, and their high throughput screening methodologies, transformation methods, selective markers, and precise gene-editing tools. We have also illustrated the applications of genetic engineering in the key areas of microalgal research, such as production of biomass, lipids, and bioactive molecules with the help of case studies along with the strategies used till date for the improvement of algal strains.

## Advancement in the Resources for Microalgal Research

### Omics Resources

#### Genomic and Transcriptomic Resources

Until 2008, only three microalgal species, namely *Chlamydomonas reinhardtii, Thalassiosira pseudonana*, and *Phaeodactylum tricornutum*, had been sequenced (Fu et al., 2019). In the last decade, revolution in “next-generation sequencing” technologies has led to the swift increase in the available number of draft as well as completed genomes of algal species (Table 1). Recently, Fu et al. (2019) have reviewed the efforts to sequence the genome of diverse group of microalgal species. The three sequencing projects, including one transcriptome sequencing and two genome sequencing projects, have been undertaken to generate the genetic resource for algal species. The transcriptome sequencing project named *Marine Microbial Eukaryote Transcriptome Sequencing Project* aimed to sequence nearly 700 marine microbial species of 17 phyla (Keeling et al., 2014). The sequence information of this dataset is available at iMicrobe Project (www.imicrobe.us/#/projects/104) and Sequence Read Archive (SRA) (BioProject PRJNA231566). Among the other sequenced transcriptomes, 140 are of marine microalgae species. Most of these sequenced species are culturable and taxonomically well-defined. Therefore, unambiguously the dataset has bias toward the gene prediction of relatively selected group of culturable isolates. Indeed, this transcriptomic data is still very helpful because it provides the extensive reference dataset for novel gene discovery and construction of computation-based metabolic models. One of the two genome sequencing projects, the *ALG-ALL-CODE*, was launched at NYU Abu Dhabi (lassb.abudhabi.nyu.edu/algallcode.php) and aimed at sequencing over 120 genomes of algal isolates belonging to several evolutionarily distinct phylum. Till date, the draft genome assemblies for 21 isolates are available in public domain, while the draft genome assemblies for 106 isolates will be available in near future. The other recently launched genome sequencing project is the 10KP, which aimed to generate genomic resource for 10,000 plants and eukaryotic microbes. Among the 10,000 genomes, at least 1,000 green algae (microalgae and macroalgae), and 3,000 photosynthetic and heterotrophic protists (majority will be of microalgae) are expected to be sequenced in 10KP genome sequencing initiative (Cheng et al., 2018). At present, around 60 algal accessions have been sequenced and their complete or draft genomes are available at “*Phytozome*” (phytozome.jgi.doe.gov) and “*The Greenhouse*” (greenhouse.lanl.gov). The complete or near to complete genome sequences for microalgae are summarized in Table 1. Altogether, these genome sequencing projects will generate a huge genetic resource for the microalgal species, which remained untapped due to the lack of information of their metabolic pathways, regulatory networks, and genetic potentials. In addition, there are three web-based resources available for algal genomics. The first database, pico-PLAZA, contains the genome information and other intuitive tools for functional genomics of 16 photosynthetic algal species (http://bioinformatics.psb.ugent.be/pico-plaza/) (Vandepoele et al., 2013). The second database is AlgaePath (http://algaepath.itps.ncku.edu.tw) that provides the details of gene expression based metabolic pathway prediction in *Chlamydomonas reinhardtii* and *Neodesmus sp*. UTEX 2219-4 (Zheng et al., 2014). The third one holds the information of gene co-expression data for two algal species (*Chlamydomonas reinhardtii* and *Cyanidioschyzon merolae*) and is available at ALCOdb (http://alcodb.jp) (Aoki et al., 2016). In addition, the random information of complete and draft genome sequence is available at JGI Genome Portal (https://genome.jgi.doe.gov) and Phytozome (https://phytozome.jgi.doe.gov). Besides the availability of robust computational methods, the complementation of the genome datasets with other omics datasets is indeed required for rational use of synthetic biology approach. For instance, the advantage of different omics datasets (genomics, proteomics, and metabolomics) and their integration for biological research is recently exemplified by sulfur-metabolic capacity of 14 diverse and representative strains of microalgae from different clades and habitats (Nelson et al., 2019).

**Table 1:** List of microalgae and diatoms with complete or near to complete genome, and the overview of reported omics studies.

**Organism (strain used for genome sequencing)**	**Genome size (Mb)**	**Conditions or aim of** ***omics*** **studies**			**Focus**	**Accession numbers and references**
		**Transcriptomic studies**	**Proteomic studies**	**Metabolomic studies/ metabolic models**		
*Auxenochlorella protothecoides* (0710)	22.92	Response to temperature and phosphate stress; trophic growth conditions; oil accumulation	Response to temperature, nitrogen and phosphorus starvation, ionizing radiation; trophic growth conditions, oil accumulation,	Response to temperature and, phosphate and nitrogen starvation, copper stress; oil accumulation, glycome profiling, trophic growth conditions / Genome scale and core metabolic model	Biofuel	PRJNA428835, PRJNA484804 (Li et al., 2013, 2014b; Gao et al., 2014a; Sibi et al., 2014; Wu et al., 2015; Park and Choi, 2018; Park et al., 2018; Vogler et al., 2018; Xing et al., 2018)
*Bathycoccus prasinos* (RCC 1105)	15.07	Normal growth conditions	-	-	Comparative analysis	PRJNA231566, https://www.imicrobe.us/#/projects/104
*Bigelowiella natans* (CCMP2755)	91.41	High light stress and small RNA profiling	Profiling of proteins targeted to plastid and peri-plastid space	-	Model Organism	GSE124831, GSE115762 (Hopkins et al., 2012)
*Botryococcus braunii* (Showa)	184.32	Response to nitrogen deprivation, high salt, cobalt enrichment, NaHCO_3_, salicylic acid, methyl jasmonate, and acetic acid	-	Response to different nutrients, growth phases; tetraterpenoid and hydrocarbons analysis / Genome scale metabolic model	Hydrocarbons and biofuels	FY358876, GES71296, SRP161189, GSE96585 (Molnar et al., 2012; Cornejo-Corona et al., 2016; Thapa et al., 2016; Blifernez-Klassen et al., 2018)
*Chlamydomonas debaryana* (NIES-2212)	120.36	-	-	Oxylipin analysis, lipid profiling in response to different light and CO_2_ levels		de los Reyes et al., 2014; Toyoshima and Sato, 2015, 2018; Yoshitomi et al., 2019
*Chlamydomonas reinhardtii* (CC-503 cw92 mt+)	120.4	Response to nutrient starvation, oxidative and heat stress, high light intensity, diurnal cycle; ciliogenesis; lipid accumulation	Response to nitrogen and sulfur starvation; exposure to high salinity, high CO_2_, dark and anoxic conditions; lipid mutant, lipid droplet proteins	Response to nitrogen starvation, dark and anoxic conditions / Genome scale and core metabolic model	Model organism	GSE17970, PRJNA379963 (May et al., 2009; Chen et al., 2010; Baba et al., 2011; Nguyen et al., 2011; Longworth et al., 2012; Mastrobuoni et al., 2012; Choi et al., 2013; Chaiboonchoe et al., 2014; Wase et al., 2014; Sithtisarn et al., 2017; Salguero et al., 2019)
*Chlorella pyrenoidosa* (FACHB-9)	56.99	Response to CO_2_ deprivation, bisphenol A, salt stress, high light stress, glucose starvation and hydroxyl radical; trophic growth conditions	Dried biomass, exposure to inhibitor of mitochondrial respiratory electron transport	Lipid profiling under copper stress and different nitrate levels / Core metabolic model	Biofuels	SRX399080, GSE40028, GSE69816, PRJNA292642, PRJNA526277 (Yang et al., 2000; Sibi et al., 2014; Liu et al., 2018b; Wan et al., 2018; Zhang et al., 2018; Duan et al., 2019)
*Chlorella sorokiniana* (1230)	58.53	Response to nitrogen deprivation, different pH, and high CO_2_	Response to inoculum sizes, light intensity and glucose concentrations, nitrogen starvation; bioactive peptide analysis	Response to high-density cultivation and UV radiation; fatty acid profiling	Biofuels	GAPD00000000, GSE98781, GCUV00000000 (Lu et al., 2013; Ma et al., 2013; Rosenberg et al., 2014; Li et al., 2015a; Chen et al., 2017; Kumar et al., 2018; Tejano et al., 2019)
*Chlorella variabilis* (NC64A)	46.16	Response to early phase of *Chlorella* virus-1 infection	-	Nitrogen deprivation and long-chain alkenes/Genome scale metabolic model	Biofuels	SRP026413 (Juneja et al., 2016; Sorigue et al., 2016)
*Chlorella vulgaris* (NJ-7)	39.08	Response to nitrogen starvation and salt stress	Response to nitrogen depletion and repletion, heterotrophic and Na induced lipid accumulation, S-nitrosylated proteome in nitrogen deplete and replete condition	Lipid profiling under copper stress, effect of graphene oxide nanomaterial, N-glycan profiling / Core metabolic model	Biofuels	LDKB00000000 (Guarnieri et al., 2011, 2013; Sibi et al., 2014; Li et al., 2015b; Ouyang et al., 2015; Henard et al., 2017; Zuñiga et al., 2018; Mocsai et al., 2019)
*Chloroidium sp*. (CF)	54.31	-		Normal growth conditions / Genome scale metabolic model	Ecological importance	Nelson et al., 2017, 2019
*Chromochloris zofingiensis* (SAG 211-14)	58	Response to nitrogen deprivation, high light; heterotrophic conditions, different growth conditions	Lipid droplets analysis	Lipid and carotenoid profiling in response to glucose	Carotenoids and fatty acids	SRP067324, GSE92515 (Wang et al., 2019c; Zhang et al., 2019)
*Coccomyxa sp*. (LA000219)	48.54	Response to arsenic treatment	-	Response to arsenic treatment	Model organism and biofuels	Koechler et al., 2016
*Coccomyxa subellipsoidea* (C-169)	48.83	Response to CO_2_ supplementation; miRNA profiling	-	Response to nitric oxide, cadmium stress, carbon source, nitrogen starvation, phytohormones	Biofuels	GSE76638 PRJNA428141 (Kováčik et al., 2015; Allen et al., 2017; Liu et al., 2018a; Wang et al., 2019e)
*Cyanidioschyzon merolae* (10D)	16.55	Response to diurnal cycle, different CO_2_ level, blue and red light, UV irradiance	Response to low temperature acclimatization; photosystem II proteins	Response to different CO_2_ level, diurnal cycle; hydrocarbon and lipid profiling in response to cyanobacterial Acyl-ACP Reductase overexpression	Model organism	GSE37673, GSE83828, GSE100372 (Krupnik et al., 2013; Rademacher et al., 2016; Nikolova et al., 2017; Miyagishima et al., 2019)
*Dunaliella salina* (CCAP 19/18)	343.7	Response to osmotic and oxidative stress, nitrogen depletion, salinity, high light; different growth phases	Response to arsenate, high salinity, high light and high bicarbonate ion level; flagella composition	Response to nitrogen starvation	Halophile, Biofuels, β-carotene and glycerol production	Katz et al., 2007; Jia et al., 2009, 2016; Gu et al., 2014; Ge et al., 2016; Lv et al., 2016; Zhao et al., 2016; Wei et al., 2017b; Wang et al., 2019d
*Emiliana huxleyi* (CCMP1516)	167.68	Response to nitrogen, sulfate and phosphorus starvation, calcium concentrations, elevated temperature and CO_2_	Response to different calcium concentration	Response to host-virus (*E. huxleyi* virus) interaction, phosphorus and nitrogen starvation; lipidomic	Coccolithophore	GSE24341, E-MTAB-2274, SRP017794, SRX756940 (Benner et al., 2013; Rokitta et al., 2014; Hunter et al., 2015; McKew et al., 2015; Wördenweber et al., 2018)
*Fistulifera solaris* (JPCC DA0580)	49.74	Response of nutrient depleted and replete conditions on lipid accumulation and its degradation	Lipid droplet proteins	-	Biofuels	DRA002404 (Nonoyama et al., 2019)
*Fragilariopsis cylindrus* (CCMP1102)	80.54	Response to temperature, high CO_2_, prolonged darkness, and nitrogen and iron limitation; small RNA profiling	Response to temperature, salinity stress, prolonged darkness, high CO_2_, iron starvation	Response to different growth phases	Psychrophile	E-MTAB-5024, GSE57987 (Lyon et al., 2011; Boroujerdi et al., 2012; Kennedy et al., 2019)
*Galdieria sulphuraria* (074W)	13.71	Response to cold acclimation	Photosystem-II analysis	-	Extremophile	PRJNA487158, GSE89169 (Thangaraj et al., 2010)
*Guillardia theta* (CCMP2712)	87.15	Small RNA profiling under light and dark conditions, mRNA splicing analysis	Response to different light intensities	-	Eukaryote endosymbiosis	GSE124831, SRR747855 (Kieselbach et al., 2018)
*Haematococcus pluvialis* (SAG 192.80)	365.78	Response to high light, salinity, iron, acetate, salicylic acid and jasmonic acid, nitrogen depletion and repletion, photooxidative stress; distinct growth phases	Cell wall protein, astaxanthin accumulation, response to high light stress, salicylic acid, and jasmonic acid	Lipid analysis, pigments and protein profiling, live single-cell analysis	Carotenoids	Wang et al., 2004; Tran et al., 2009; Peled et al., 2011; Gu et al., 2014; Recht et al., 2014; Su et al., 2014; Gao et al., 2016; Baumeister et al., 2019; Luo et al., 2019
*Helicosporidium sp*. (ATCC 50920)	12.37	Transition from free-living organism to obligate intracellular parasite	-	-	Parasite	Pombert et al., 2014
*Klebsormidium nitens* (NIES-2285)	104.21	Response to auxin treatment and cold stress	-	Response to cold stress	Tolerance to UV and harsh conditions	PRJDB4958, PRJNA500592 (Nagao et al., 2008)
*Micromonas commoda* (RCC299)	21.11	Response to different light regimes and ultra-violet light stress	Response to chronic phosphate limitation and subsequent relief, high light and UV-radiation	-	Marine phytoplankton	Cuvelier et al., 2017; Guo et al., 2018
*Micromonas pusilla* (CCMP1545)	21.96	Response to phycodnavirus MpV-SP1 infection, phosphate deplete and replete, day-night cycle	Phosphate deplete and replete condition, day-night cycle	Response to phosphate deplete and replete condition; different growth phases,	Marine phytoplankton	PRJNA422663 (van Baren et al., 2016; Waltman et al., 2016; Kujawinski et al., 2017)
*Micromonas sp*. (ASP10-01a)	19.58	Normal growth conditions	-	-	Marine phytoplankton	van Baren et al., 2016
*Monoraphidium neglectum* (SAG 48.87)	69.71	Nitrogen deprivation	-	-	Biofuels	PRJNA221625 (Jaeger et al., 2017)
*Nannochloropsis gaditana* (CCMP1894)	30.86	Response to light intensity regimes and nitrogen replete and deplete condition	Fresh and atomized biomass	Response to light intensity regimes and nitrogen deprivation / Genome scale metabolic model	Biofuels	Radakovits et al., 2012; Sorigue et al., 2016; Ajjawi et al., 2017; Shah et al., 2017; Fernandez-Acero et al., 2019; Patelou et al., 2020
*Nannochloropsis limnetica* (CCMP505)	33.51	-	-	Nitrogen deprivation	Biofuels	Sorigue et al., 2016
*Nannochloropsis oceanica* (LAMB2011)	29.26	Response to different CO_2_ levels, phosphorus and nitrogen limitation, light and dark cycle, fresh water acclimation; transition from quiescence to autotrophy	Response to long-term nitrogen starvation, low CO_2_; single-cell-level phenotypic heterogeneity	Response to osmotic downshift and nitrogen depletion	Biofuels	Dong et al., 2013; Pal et al., 2013; Sorigue et al., 2016; Poliner et al., 2018; Chen et al., 2019; Wei et al., 2019
*Nannochloropsis oculate* (CCMP525)	26.27	-	Nitrogen deprivation, cadmium stress	Nitrogen deprivation	Lipids and protein content	Kim et al., 2005; Sorigue et al., 2016; Tran et al., 2016
*Ostreococcus lucimarinus* (CCE9901)	13.2	-	-	Genome scale metabolic model	Small genome	Krumholz et al., 2012
*Ostreococcus tauri* (RCC4221)	13.03	Response to OtV5 virus infection, light and dark cycle, iron limitation, and high light; life cycle stages	Phosphoproteome in response to casein kinase 2, light dark cycle	Glycerolipid profiling under nutrient deprived condition, diurnal variations, nitrogen deprivation / Genome Scale metabolic model	Small genome	Krumholz et al., 2012; Martin et al., 2012; Hindle et al., 2014; Le Bihan et al., 2015; Lelandais et al., 2016; Sorigue et al., 2016; Degraeve-Guilbault et al., 2017; Hirth et al., 2017
*Parachlorella kessleri* (NIES-2152)	59.18	Response to salt stress and sulfur deplete and replete	Salt stress	Nitrogen, sulfur and phosphorus deprivation		Ota et al., 2016a,b; Shaikh et al., 2019; You et al., 2019
*Phaeodactylum tricornutum* (CCAP 1055/1)	27.45	Response to nitrogen, iron, carbon and phosphorus deprivation, cadmium stress, mixotrophic growth, grazing stress, different light intensities, and regimes, salicylic acid; non-coding microRNA	Response to nitrogen limitation, oxidative and dark stress; phosphoproteomics under high light, nitrogen, and iron deficiency	Response to blue and red light, nitrogen and phosphorus deprivation; glycerolipid profile; mixotrophic growth / Genome scale and core metabolic model	Model organism	PRJEB11970, SRX648639 (Chen et al., 2014; Ge et al., 2014; Jungandreas et al., 2014; Rosenwasser et al., 2014; Yang et al., 2014; Abida et al., 2015; Alipanah et al., 2015; Feng et al., 2015; Bai et al., 2016; Longworth et al., 2016; Sorigue et al., 2016; Yoneda et al., 2016; Villanova et al., 2017; Remmers et al., 2018; Smith et al., 2019)
*Picochlorum sp*. (SENEW3 / DOE 101)	13.39 / 15.25	Response to salinity stress and high temperature	-	-	Biofuels	PRJNA245752, PRJNA389600
*Scenedesmus sp*. (ARA3, ARA)	93.24	Response to phosphorus and nitrogen starvation, lipid accumulation	Response to salinity stress; lipid accumulation	Response to salinity and arsenic stress; lipid accumulation	Biofuels	PRJNA428298 (Chu et al., 2011; Arora et al., 2018, 2019; Wang et al., 2019b)
*Scenedesmus obliquus* (UTEX393)	107.72	Response to diurnal changes and nC_60_; wild type and starch less mutant comparison	Thylakoid membrane proteome, toxicity of silver nanoclusters	Response to nC_60_ and silver nanoparticles; different photoperiod and growth phases	Lipid and biomass	E-MTAB-7009 (Kantzilakis et al., 2007; Du et al., 2017; Zhang et al., 2017; Vendruscolo et al., 2019; Wang et al., 2019a)
*Symbiodinium minutum* (Mf 1.05b.01)	609.48	Diurnal cycle, cultured, and freshly isolated cells	-	Response to acidification	Coral symbiont	PRJNA544863 (Jiang and Lu, 2019)
*Symbiodinium microadriaticum* (CCMP2467)	808.2	Response to different temperature, dark, and salinity stress; normal growth conditions, miRNA profiling	-	Response to environmental variation	Coral symbiont	GSE47373, GSE47372 (Klueter et al., 2015; Aranda et al., 2016)
*Tetraselmis striata* (LANL1001)	227.95	Normal growth	-	-		PRJNA231566, https://www.imicrobe.us/#/projects/104
*Thalassiosira oceanica* (CCMP1005)	92.18	Response to iron and copper	Response to iron and copper; extracellular superoxide production	-	Model organism	PRJNA382002, SRA045825 (Lommer et al., 2012; Diaz et al., 2019)
*Thalassiosira pseudonana* (CCMP1335)	32.44	Response to nitrogen and phosphorus deprivation, salinity, light intensity, triphenyltin chloride, silicon, CO_2_ levels, source of light, and nitrogen	Response to nitrogen and phosphorus starvation, light intensity, salinity, triphenyltin chloride, CO_2_ levels, silicon, micronutrients deficiency, benzo(a)pyrene, *K. brevis* allelopathy; composition of nano- and micropatterned biosilica cell wall, mitochondrial and plastid proteome	Response to phosphate deplete and replete condition, cobalamin scarcity; *K. brevis* allelopathy	Model organism	Carvalho and Lettieri, 2011; Dyhrman et al., 2012; Du et al., 2014; Kettles et al., 2014; Kustka et al., 2014; Luo et al., 2014; Poulson-Ellestad et al., 2014; Yi et al., 2014; Jian et al., 2017; Kujawinski et al., 2017; Chen et al., 2018; Heal et al., 2019; Schober et al., 2019
*Trebouxia gelatinosa* (LA000220)	61.73	Response to dehydration and subsequent rehydration	-	-	Colonization through symbiosis	PRJNA213702
*Volvox carteri* f. *magariensis* (Eve)	137.68	Response to low dose of UV-B radiation; somatic and reproductive cells	-	-	Multicellular alga, model organism	E-MTAB-5691 and GSE104835
*Yamagishiella unicocca* (NIES-3982)	134.24	Normal growth condition	-	-	Multicellular alga, model organism	PRJNA532307

#### Proteomic Resources

The quantitative data of protein expression under different experimental conditions is advantageous for better understanding of regulatory pathways, which differ at the post-transcriptional level. Since, several studies failed to give a high correlation between transcriptomic and proteomic data (Haider and Pal, 2013), the availability of quantitative proteomic and transcriptomic data under defined experimental condition will provide strategic insights for strain improvement in microalgae. In particular, several analyses have been performed to identify the proteome dynamics and the corresponding transcriptome analysis. However, this was mainly focused to understand the lipid metabolism in model and/or oleaginous microalgae with potential of biofuel production (Table 1). The literature mining shows that the majority of proteomics studies were performed under experimental conditions, including nitrogen starvation, copper deprivation, light intensity regimes, heterotrophic cultivation, and salt stress (Table 1). The majority of differentially abundant proteins were found to have functions in metabolic pathways related to fatty acid and lipid metabolism, carbohydrate metabolism, photosynthesis, and cell structure integrity and maintenance. In addition, the large numbers of algal proteins have been predicted through genomic sequence analysis and the information is available at the Uniprot (https://www.uniprot.org) and Protein Data Bank archive (https://www.rcsb.org). In an attempt to comprehensively cumulate the structural, physicochemical, and functional information of algal proteome, the non-redundant protein database of 31 algal species was developed and is available at Algal Protein Annotation Suite (Alga-PrAS) (Kurotani et al., 2017).

#### Metabolomics and Metabolic Models

The metabolites are the intermediate or end products of the cellular regulatory processes that are implicated through the transcriptome and proteome, and thus represent the cellular response to the stimulus. Some metabolites are also involved in the regulation of cellular responses by regulating the activity of enzymes involved (Wegner et al., 2015). Thus, information of metabolic profile in response to the experimental conditions may help to target the processes or pathways, which could be helpful in metabolic-engineering of microalgal strains. The quantitative and qualitative analysis of metabolites is now fairly possible even though they have a wide variation in chemical properties, such as polarity, charge, solubility, volatility, and molecular weight. This has become possible due to the advances in non-targeted metabolite profiling and its platforms, such as capillary electrophoresis-mass spectrometry, gas chromatography-mass spectrometry, liquid chromatography-mass spectrometry, Fourier transform ion cyclotron resonance-mass spectrometry, and nuclear magnetic resonance spectroscopy. Similar to the transcriptome and proteomic studies in microalgae, the majority of metabolomic (though only few untargeted metabolic studies reported) studies were also focused on lipid metabolism under various environmental conditions (Table 1). Recently, the potential of single-probe mass spectrometry technology has been demonstrated for near *in-situ* analyses of single cell of *Scrippsiella trochoidea* under nitrogen starving and light vs. dark conditions to analyze the lipid content and lipid profile (Sun et al., 2018). This single-cell-targeted metabolomics may prove to be instrumental in the future algal research, since it reduces the chances of experimental artifacts and confounds, thereby minimizing the cell to cell metabolic variability. Unlike genome and transcriptome databases, unfortunately, no dedicated database is available for the microalgal metabolomics. Although the attempts were made to reconstruct genome-scale metabolic models at system level, they are based on the information of the genome, transcriptome, and scarcely available experimental data. For organisms like *C. reinhardtii, Chlorella* spp., *P. tricornutum*, and some blue-green algae (cyanobacteria), the genome-scale metabolic models are available. The core metabolic models and genome-scale system-level metabolic networks available for different microalgal species are given in Table 1. In addition, some databases, such as KEGG (https://www.genome.jp/kegg/pathway.html), Reactome (https://reactome.org), and Metacyc (https://metacyc.org) contain predicted and experimentally proven metabolic network information and can be explored for predictive and integrative biology in microalgae. The information available through the genetic characterization of cellular pathways, and high throughput genome-scale studies under different experimental conditions, is contributing toward the refinement of metabolic models for system-level analysis of biological processes.

### Mutant Resources for Microalgae

The mutant library for an organism is the best available tool to accelerate the functional characterization of enormous set of uncharacterized genes for better understanding of fundamental biological processes. The potential of such mutant libraries has been exemplified by those that are available for organisms such as *Saccharomyces cerevisiae* (www.sequence.stanford.edu/group/yeast_deletion_project/deletions3) and *Arabidopsis thaliana* (www.arabidopsis.org/portals/mutants/index.jsp). The mutant libraries are instrumental in the reverse genetic studies. However, generating such libraries for microalgae is limited by the lack of efficient transformation and genetic manipulation protocols (discussed in later sections). The insertional mutagenesis through random non-homologous end-joining is the method of choice to generate the mutant libraries. Till date, only two genomewide random insertion mutant libraries have been generated for *C. reinhardtii* using the insertional mutagenesis approach. The first collaborative project, Chlamydomonas Library Project (CLiP) was launched in 2010 by Jonikas (now at Princeton University, USA) and Grossman at Carnegie Institution for science (USA), Fitz-Gibbon (University of California Los Angeles, USA), and Lefebvre (University of Minnesota, USA) to generate the genome-scale insertional mutant library for *C. reinhardtii*. The mutants from this library have been released for the research community and other stakeholders on periodic basis. The complete library featuring more than 62,000 mutants that covers 83% of nuclear protein-encoding genes is now available at Chlamydomonas Resource Center (www.chlamycollection.org/products/clip-strains). Importantly, the mutants in this library are fully mapped for insertion sites and indexed with unique DNA barcode for high-throughput screening of pooled mutants for a particular trait or biological process (Li et al., 2016b, 2019). Similarly, the Huang group at Institute of Hydrobiology, China, generated another insertional mutant library of *C. reinhardtii* with ~150,000 insertional mutants (Cheng et al., 2017). Although this library contains higher number of mutants than that of CLiP, the list of mutants and their mapping information is not available in public domain. In addition, a non-indexed insertional mutant library of *C. reinhardtii* with ~49,000 mutants was also developed and is available for the scientific community at Chlamydomonas Resource Center (http://chlamycollection.org). The potential utility of these mutant libraries can be attributed to the discovery of novel candidate genes involved in biological and physiological processes, such as photosynthesis, lipid biosynthesis, and intraflagellar transport in microalgae (Dent et al., 2015; Li et al., 2016b, 2019).

In addition to the insertional mutagenesis, the mutagenic agents are being regularly used to generate the mutant strains with desired traits. Several attempts have been made using forward genetic approach to characterize the genes involved in the molecular pathways targeting a desired trait. Since the *C. reinhardtii* is considered as premier reference organism for understanding the basic algal metabolism and biological processes, most of the forward genetic screens have been performed in this model organism. These forward genetic screening in *C. reinhardtii* and some other model microalgae have been performed mostly to identify the genetic factors responsible for desirable traits, such as higher biomass and cell culture density (Thung et al., 2018), enhanced lipid content (Cagnon et al., 2013; Lee et al., 2014), or to understand the basic cellular processes such as photosynthesis (Dent et al., 2015; Li et al., 2019), non-photochemical quenching (Schierenbeck et al., 2015), lipid metabolism (Li et al., 2016b; Schulz-Raffelt et al., 2016; Cheng et al., 2017), and flagellar responses (Hilton et al., 2016; Cheng et al., 2017). In an integrative approach, the *P. tricornutum* mutants with enhanced carotenoid biosynthesis were subjected to genome-scale metabolic network simulation to identify the metabolic reactions that are highly correlated with the carotenoid biosynthesis (Yi et al., 2018). This study exemplified the use of system-biology approach to target the key pathway(s) that should be considered during bioengineering in diatoms. Recently, using a modified approach named as bulked mutant analysis and bulked mutant RNA sequencing, the single nucleotide polymorphisms and indels were identified that are associated to the growth-related genes in *Nannochloropsis oceanica* (Liang et al., 2019). These methods of forward genetic screen have the potential to facilitate the genetic investigation of diverse microalgae with various desirable traits.

### High-Throughput Screening Methodologies for Microalgae

The previous section reviewed various genomic and mutant resources that are available for the microalgae research. The resources for microalgal forward genetics have the potential to revolutionize the identification of mutants with desired traits, however limited to availability of the rapid screening methods. Moreover, the screening of microalgae natural pools to identify functional components is low due to the lack of effective rapid and high-throughput analysis tools (Lee et al., 2013). In addition, this also limits our capacity for the real-time monitoring of process for target compound production using microalgae. To enrich the mutants capable of accumulating high lipid content, Sharma et al. (2015) developed and validated a high-throughput work flow strategy based on *in-situ* analysis of lipid bodies using confocal Raman microscopy combined with fluorescence activated cell sorting (FACS). A precise and efficient Raman platform was developed to distinguish the contrasting features of lipids such as chain length and saturation level in lipid-expressing cells generated through UV mutagenesis. Terashima et al. (2015) introduced another high-throughput advanced technique, named *Chlamydomonas* high-lipid sorting (CHiLiS), which enables to isolate mutants with high lipid content. CHiLiS is based on the fact that Nile Red (lipid detecting dye)-stained lipid pools were enriched by using FACS. In this method, the staining extent was raised to a certain level for increasing the enrichment tendency without interfering with the cell's viability. These high-throughput methods have the potential of selecting the mutant strains that can be used either for the understanding of molecular basis of high lipid accumulation or engineering of microalgae for maximizing the production of lipids. Based on the staining of lipid bodies with fluorescent dyes, several high throughput systems are available commercially. Semi-automated QPix^TM^ 400 Series system from Molecular Devices is one such example (https://www.moleculardevices.com/sites/default/files/en/assets/brochures/biologics/qpix-400-systems). The Fourier transform infrared spectroscopy also demonstrated its sensitivity to screen mutants of *C. reinhardtii* for variation in their lipid and carbohydrate profile under specific nutrient stress conditions (Bajhaiya et al., 2016). Based on this screening, nutrient starvation response genes (*PSR1, SNRK2*.1, and *SNRK2*.2) with possible role in lipid and starch accumulation were identified.

In an another approach, to isolate the algal cells with superior photosynthetic activity, the high-throughput microfluidics were used in the microalgal selection process (Kim et al., 2016). This system used the strong positive relationship between phototaxis and photosynthetic efficiency, where the competitive phototactic response was employed to isolate the highly photosynthetic efficient strains at the single-cell level using a microfluidic system. Also, the putative candidate genes related to the transcriptional regulation (JGI Chlre4, protein ID: 525919, 516641, 513996), cellular metabolism (519327, 523869, 515661) signal transduction (516786), flagellar function (518826), and membrane transport (protein ID; 516748, 516786, 513005, 520695, 512634) were identified, that might have some role in enhanced photosynthetic activity and phototactic response in mutant strains. The putative candidate genes identified in this study may be cataloged for their use in microalgal strain engineering strategies. Even after the identification of photosynthetic efficient microalgal strains, optimization of the light conditions remains critical to augment system efficiency. Recently, a novel high-throughput screening system was developed by Sivakaminathan et al. (2018), which simulates fluctuating light regimes in mass cultures. This high-throughput miniaturized light system is capable of screening up to 18 different combinations of light regime and up to 1,728 conditions to evaluate species-specific light conditions for maximum photosynthetic efficiency and productivity.

For the screening of biopigments accumulation, a 96-well microplate-based high-throughput assay was developed to identify *P. tricornutum* mutants with high carotenoid content (Yi et al., 2018). The assay was based on the fact that fluorescence intensity of chlorophyll a and neutral lipids (stained with fluorescence dye) has a significant correlation with the carotenoid content during exponential growth phase of *P. tricornutum*. Generally, the *in-situ* optical detection-based methods fail to provide detailed information on the pigment composition in microalgae because of the possible overlapping of absorbance and emission spectra of various pigments. In such cases, the extraction and subsequent detection is the only method of choice. However, the extraction of a particular pigment type is a time-consuming multi-step process that also required a suitable extraction solvent to effectively extract the pigment. A rapid and reliable microwave-assisted extraction and subsequent detection of microalgal pigment using relevant method could be helpful in developing high-throughput screening platform for microalgal pigments (Pasquet et al., 2011). An enzyme-linked immunosorbent assay (ELISA) on microtiter platform was developed by Jirásková et al. (2009) to detect the presence of phytohormones, such as abscisic acid, indole-3-acetic acid, cis- and trans-zeatin, and isopentenyladenosine in microalgae. This high-throughput application of ELISA-based microtiter platform can be extrapolated to the other bioactive compounds if suitable antibodies and/or antigens are available. Likewise, a simple and inexpensive high-throughput bioassay was developed to screen the algal mutants or isolates producing high H_2_ under saturating light intensity (Wecker and Ghirardi, 2014). The screening assay used the agar overlay of *Rhodobacter capsulatus* bacteria carrying a green fluorescent protein that responds to H_2_ produced by single algal colony. Among the other high-throughput screening methods, the phenotype microarray technologies have also shown promise to screen-defined metabolic activities in response to array of different drugs, chemicals, and metabolites (www.biolog.com).

### Genetic Engineering in Microalgae

#### Transformation Technologies and Selectable Markers

The first nuclear transformation of *C. reinhardtii* using polyethylene glycol or poly-L-ornithine was demonstrated in early 1980's. Here, the complementation of arginine-requiring, cell-wall deficient mutant was performed through successful integration of yeast *arg4* locus (Rochaix and Dillewijn, 1982). In the late 1980's, the successful stable nuclear transformation in *C. reinhardtii* was demonstrated using the biolistic transformation approach to deliver the native genes to complement auxotrophic growth in mutants (Debuchy et al., 1989; Kindle et al., 1989; Mayfield and Kindle, 1990). Later in the 1990's, the success of glass bead agitation and electroporation were demonstrated, where the later was found to be the most efficient method to transform the nuclear genome of *C. reinhardtii* (Kindle, 1990). The droplet electroporation on microfluidic chip was found to have threefold higher transformation efficiency than the electroporation cuvettes (Qu et al., 2012). In addition, the use of other methods by employing silicon carbide whiskers (Dunahay, 1993), *Agrobacterium tumefaciens* (Kumar et al., 2004), and nanoparticles (Kim et al., 2014) have been also demonstrated to successfully transform the nuclear genome of *C. reinhardtii*. The methods for the nuclear transformation in other microalgal species such as *Phaeodactylum, Nannochloropsis, Dunaliella*, and *Haematococcus* are available (Table 2). The various transformation techniques and the selectable markers used for the screening of transformants, and mainly includes the use of antibiotic, herbicide resistance, and auxotrophic markers are listed in Table 2. The evolutionary divergence of the cellular machinery in microalgae, however, limits the use of existing plant or other microbe-based selectable markers for selection purposes. For instance, the trait stacking in the industrially important microalgae “*Nannochloropsis*” through genetic engineering is mostly limited by the availability of selectable markers (Verruto et al., 2018). The use of auxotrophic selection marker is mostly a desirable trait; however, a pre-requisite that the strain to be transformed must be auxotrophic mutant for the selectable marker, which may sometimes interfere with the experimental setup.

**Table 2:** List of microalgae and the molecular tool and techniques available for their genetic engineering.

**Organism**	**Genetic tools and techniques**			**References**
	**Transformation**	**Genetic manipulation**	**Selectable markers**	
*Botryococcus braunii*	Electroporation	Gene integration and expression	Antibiotic: *aphVIII*	Berrios et al., 2016
*Chlamydomonas reinhardtii*	Biolistic, glass bead agitation, electroporation, and agrobacterium-mediated	Gene expression, RNA interference, gene-editing using ZFNs and CRISPR	Antibiotic: *aphVIII, aphVII, nptII, addA, tetX, hph*, and *ble*. Autotrophic: *arg* and *trp*. Herbicide: 2-fluoroadenin resistance	Kim and Cerutti, 2009; Greiner et al., 2017; Mini et al., 2018
*Chlorella pyrenoidosa*	Electroporation	Gene integration and expression	Antibiotic: *nptII*	Run et al., 2016
*Chlorella sorokiniana*	Biolistic	Gene integration and expression	Autotrophic: *nr*	Dawson et al., 1997
*Chlorella vulgaris*	Electroporation, glass bead agitation, and agrobacterium-mediated,	Gene integration and expression	Antibiotic: *nptII* and *aphVII*	Cha et al., 2012; Muñoz et al., 2018
*Chromochloris zofingiensis*	Biolistic	Gene integration and expression	Herbicide: norflurazon-resistance	Liu et al., 2014
*Coccomyxa* sp.	Biolistic and electroporation	Gene integration and expression, gene-editing using CRISPR	Autotrophic: *umps*	Kasai et al., 2018; Yoshimitsu et al., 2018
*Coccomyxa subellipsoidea*	Electroporation	Gene integration and expression	Antibiotic: *hptII*	Kania et al., 2019
*Cyanidioschyzon merolae*	PEG-mediated	Gene integration and expression, RNAi	Antibiotic: *cat* Auxotrophic: *ura*	Ohnuma et al., 2009; Sumiya et al., 2015; Fujiwara et al., 2017
*Dunaliella salina*	Electroporation, biolistic, glass beads agitation, and agrobacterium-mediated	Gene integration and expression	Antibiotic: *aphVII* and *nptII* Herbicide: *bar* Auxotrophic: *nr*	Li et al., 2007; Radakovits et al., 2010; Srinivasan and Gothandam, 2016
*Fistulifera solaris*	Biolistic	Gene integration and expression	Antibiotic: *nptII*	Muto et al., 2013
*Gonium pectorale*	Biolistic	Gene integration and expression	Antibiotic: *aphVIII*	Lerche and Hallmann, 2009
*Haematococcus pluvialis*	Biolistic	Gene integration and expression	Antibiotic: *aadA* Herbicide: norflurazon resistance	Steinbrenner and Sandmann, 2006; Yuan et al., 2019
*Monoraphidium neglectum*	Electroporation	Gene integration and expression	Antibiotic: *aphVII*	Jaeger et al., 2017
*Nannochloropsis gaditana*	Electroporation	Gene integration and expression, gene-editing using CRISPR	Antibiotic: *aphVII, nptII* and *BSD*	Ajjawi et al., 2017
*Nannochloropsis limnetica*	Electroporation	Gene integration and expression	Antibiotic:*aphVII* and *nptII*	Chen and Hu, 2019
*Nannochloropsis oceanica*	Electroporation	Gene integration and expression, RNAi, gene-editing using CRISPR	Antibiotic: *sh ble* and *nptII*	Li et al., 2014a; Poliner et al., 2018; Osorio et al., 2019
*Nannochloropsis oculata*	Electroporation	Gene integration and expression	Antibiotic: *sh ble*	Li et al., 2014a
*Ostreococcus tauri*	Electroporation and PEG-based	Gene integration and expression	Antibiotic:*nptII* and *neo*	van Ooijen et al., 2012; Sanchez et al., 2019
*Parachlorella kessleri*	Biolistic and agrobacterium-mediated	Gene integration and expression	Antibiotic: *nptII* and *aadA*	Rathod et al., 2013
*Phaeodactylum tricornutum*	Biolistic, electroporation, and bacterial conjugation	Gene integration and expression, gene-editing using MNs, CRISPR, and TAELNs	Antibiotic: *nat, sat-1, addA, sh ble* and *cat* Autotrophic: *ura* Herbicide: 5-fluoroorotic acid and 2-fluoroadenine resistance	Daboussi et al., 2014; Serif et al., 2018; Sharma et al., 2018
*Scenedesmus obliquus*	Electroporation	Gene integration and expression	Antibiotic: *cat*	Guo et al., 2013
*Symbiodinium microadriaticum*	silicon carbide whiskers	Gene integration and expression	Antibiotic:*nptII* and *hpt*	Te et al., 1998
*Thalassiosira pseudonana*	Biolistic and bacterial conjugation	Gene integration and expression, gene-editing using CRISPR	Antibiotic: *nat* and *sat-1*	Karas et al., 2015
*Volvox carteri* f. *magariensis*	Biolistic	Gene integration and expression, gene-editing using CRISPR	Antibiotic: *hpt* and *BSD* Autotrophic: *nr*	Ortega-Escalante et al., 2019a,b

Despite the recent advancement in the transformation technologies, the microalgae transformation is still facing the problem of low efficiency, except in *Chlamydomonas* when compared to the plant system. In an advancement, the development of nuclear episomal vector to transform diatoms *via* conjugation-based method that directly deliver the vector from *E. coli* to diatom provides an efficient method for diatom transformation (Karas et al., 2015). This method offers several advantages over the conventional transformation methods such as capacity to deliver large DNA fragments (may be multiple genes from a pathway), stable self-replication of episomal vector (due to presence of yeast-derived regulatory sequences, CEN/ARS), loss of transgene upon removal of selection pressure, and low possibility of positional or epigenetic effects (Doron et al., 2016). Recently, the application of conjugation-based method in CRISPR/cas9 mediated genome-editing of *Pt MYBR1* gene in *P. tricornutum* (Sharma et al., 2018) and nitrate reductase gene (*NR*) in *Nannochloropsis oceanica* (Poliner et al., 2019) has been demonstrated to generate transgene-free mutants. Although 20–100 times higher transformation efficiency and rapid transformant appearance was observed in the conjugation-based method, there was a significant delay in the appearance of mutants in the positive transformants (Sharma et al., 2018). The plausible explanation for this delayed mutant appearance was attributed to the lower Cas9 expression due to higher rate of cell division in conjugatively transformed cells. In addition, the episomal vector system adapted for diatom was able to transform the green oleaginous microalgae *Acutodesmus obliquus* and *Neochloris oleoabundans* through bacterial conjugation (Muñoz et al., 2019). Although the transformation efficiency was sufficiently higher as compared to the biolistic-based vector delivery system, this application of diatom adapted episomal vector system in other microalgae has some limitations that are discussed in the following section.

#### Genome-Editing

Over the years, significant progress has been made to improve the catalog of available tools for genetic engineering in microalgae, with the ultimate aim to improve the feasibility of microalgae as a model organism for scientific and/or industrial applications. In the past decade, the gene-editing tools such as zinc-finger nucleases (ZFNs), meganucleases (MNs), transcription activator-like effector nucleases (TALEN), and clustered regularly interspaced short palindromic repeats (CRISPR/Cas9) have been emerged as the efficient tools for genome-editing in many organisms (Razzaq et al., 2019). All these tools are able to introduce a double-strand break at targeted DNA sequence that can be further repaired *via* either non-homologous end-joining (may disrupt gene through mutations) or homologous recombination (may insert or replace gene with exogenous donor DNA) (Jeon et al., 2017). The CRISPR is often used interchangeably to the term genome-editing; however, the ZFNs and TALENs were among the first molecular tools available for the genome-editing. The applicability of these tools largely depends on the factors such as cost, complexity, and ability to cause multiple edits simultaneously. Among the others, the CRISPR/cas9-mediated genome-editing system now became the state-of-the-art tool due to its simplicity and versatility.

The initial reports of gene-editing in microalgae were from ZFN-mediated genome-editing. Sizova et al. (2013) and Greiner et al. (2017) used engineered ZFNs to target the *COP3* and *COP4* genes in *C. reinhardtii*. However, the efficiency of the ZFNs was only observed in the tailored model strain of *C. reinhardtii*. In addition, it was also suggested that ZFNs prefer the homology-directed repair when supplied with larger donor DNA (>750 bp) for the clean and predictable gene modification. Beside the recent developments in the ZFN technology, the most challenging task is to create unique ZFNs with high specificity and affinity toward the target sites. This requires the validation of ZFNs using gene targeting selection system before conducting the actual experiment (Sizova et al., 2013). Meanwhile, the use of MNs and TALENs was also demonstrated to target the *uridyl diphosphate* (*UDP*)*-glucose pyrophosphorylase* in *P. tricornutum* for enhanced lipid accumulation (Daboussi et al., 2014). The use of TALENs for the disruption of urease gene through homologous recombination has been successfully achieved in *P. tricornutum* (Weyman et al., 2015). Similarly, in an attempt to evaluate the use of uridine monophosphate (UMP) synthase as an endogenous positive selectable marker for DNA-free genome editing, Serif et al. (2018) used TALEN to generate knock-out mutants of *UMP synthase* gene in *P. tricornutum*. Although the efficiency of the gene disruption using TALENs was quite low (only 16%), the applicability of TALENs for gene-editing in microalgae has been established. However, though the use of TALENs for gene-editing has been exemplified in several organisms, no report has been observed till date in *Chlamydomonas*. The functioning of transcription activator-like effectors (TALEs) has been established in *Chlamydomonas* to induce the expression of endogenous genes, *ARS1* and *ARS2* through the binding of gene-specific artificially designed TALEs to the promoter region of the targeted genes (Gao et al., 2014b). This study indicates that the TALEs coupled to nuclease(s) can (TALENs) be used as one of the approaches to target the gene-editing in *Chlamydomonas*.

The successful use of CRISPR/cas9 system in microalgae species was first demonstrated by Jiang et al. in *C. reinhardtii* (Jiang et al., 2014). In this study, four genes were successfully edited through the expression of codon-optimized *Cas9* gene and corresponding single guide RNA (sgRNA). However, the constitutive expression of Cas9 shows cytotoxic effect in *C. reinhardtii* that reduce the cell viability of transformants (Ng et al., 2020). Therefore, the transient delivery of *in-vitro* assembled Cas9/sg RNA ribonucleoprotein (RNP) complex *via* electroporation is a promising methodology to efficiently edit genes in *C. reinhardtii* without the cytotoxic effect of Cas9, and this approach was established recently (Shin et al., 2016a; Baek et al., 2016a). The use of Cas9/sgRNA-RNP-complex-mediated approach could exempt the genome-edited microalgae from the regulations of genetically modified organism (GMO) regulations, since it does not involve the integration of foreign DNA (*cas9* gene) in the host genome. In addition, the Cas9/sgRNA RNP complex further reduces the off-target effects and is less cytotoxic to the cells because of transient expression of cas9, thus improving the efficiency of gene-editing. In an effort to improve the efficiency of CRISPR/Cas9 system in *C. reinhardtii*, Jiang and Weeks (2017) employed gene-within-a-gene methodology that uses hybrid *Cas9* gene containing an artificial intron having sgRNA gene. Although the hybrid cas9 system was functional in *Chlamydomonas*, the improvement in the efficiency of gene editing was only marginal. A higher editing-efficiency of up to 9 and 3.3% in *Chlamydomonas* was observed by Greiner et al. (2017) after using a *Cas9* gene from *Staphylococcus aureus* and *S. pyogenes*, respectively. Recently, Guzmán-Zapata et al. (2019) used transient expression of *S. pyogenes* cas9 to disrupt the *atp9* gene in *Chlamydomonas* with efficiency of up to 30% on preselected 2-fluoroadenine resistant colonies. This approach of pre-selection based on the editing of selectable marker gene could also be used for the multiplexed editing. In another approach, an ortholog of cas9, Cpf1 was used in single-step co-delivery of CRISPR/Cpf1 RNP complex along with single-stranded DNA repair template, and this approach resulted in ~10% efficiency for precise gene-editing in *C. reinhardtii* (Ferenczi et al., 2017). Using dcas9 (dead cas9, nuclease defense), the functioning of a variant of CRISPR, named as CRISPRi (CRISPR interference) was also established in *C. reinhardtii* through downregulation of *PEPC1* expression to enhance the lipid content (Kao and Ng, 2017).

Besides *Chlamydomonas*, the adaptability of the CRISPR system was also successfully demonstrated for another model marine microalgae *P. tricornutum*. Using codon-optimized *S. pyogenes cas9*, the disruption of *P. tricornutum CpSRP54* gene with 31% efficiency indicates that, unlike *Chlamydomonas*, the Cas9 constitutive expression is not likely to be toxic for diatoms (Nymark et al., 2016). Recently, Sharma et al. (2018) compared the effect of constitutive and transient expression of cas9 on editing frequency and stability of mutant lines generated through biolistic and bacterial conjugation, respectively. Although the efficiency of CRISPR-induced targeted mutations were similar for both methods, the use of conjugation-based episomal CRISPR/Cas9 system is capable of avoiding re-editing of mutant lines caused by constitutive expression of Cas9 in the progeny (Sharma et al., 2018; Slattery et al., 2018). Intriguingly, the simultaneous knock-out of multiple genes has also been demonstrated in *P. tricornutum* through the delivery of Cas9/sgRNA RNP complex (Serif et al., 2018). In addition, the CRISPR/cas9 system was also able to edit urease gene in another diatom, *T. pseudonana* with up to more than 60% of disruption efficiency (Hopes et al., 2016). The application of CRISPR system on industrially important oleaginous marine microalgae *N. oceanica* was first demonstrated through the disruption of nitrate reductase gene (Wang et al., 2016), however with a very low efficiency of nearly 1%. Later, the cas9 editor line of *N. gaditana* was developed that constitutively expressed the cas9 and was used for editing of targeted transcription factor genes with high efficiency range of up to 78% (Ajjawi et al., 2017). In the recent past, various strategies have been successfully applied for gene-editing in several microalgal species. However, the above literature shows the inconsistency in the editing-efficiency of CRISPR system across the microalgal species and is still a concern. Thus, the identification of novel or optimized nucleases that may prove to be useful in gene-editing in microalgae is required. Moreover, the constitutive expression of Cas9 (or other nucleases) may sometime induce the undesired re-editing of the mutant lines (Slattery et al., 2018). In this context, the episomal-vector system has the advantage of transient Cas9 (or other nucleases) expression that can prevent re-editing of mutant lines, which is a common complication associated with the constitutive expression of Cas9. Moreover, the elimination of episomal CRISPR/Cas9 vector from the host upon removal of selection pressure makes the mutant lines be considered as non-transgenic. In contrast to diatom, using similar vector system, recently Muñoz et al. (2019) were not able to rescue the episomal plasmids from positive transformants of green oleaginous microalgae *Acutodesmus obliquus* and *Neochloris oleoabundans*. Moreover, the continuous subculturing in the selection-free medium was not sufficient to remove the episomal vector. This indicates the possible chromosomal integration event even in the bacterial conjugation-based episomal vector delivery. Therefore, the episomal maintenance of delivered plasmids in microalgae other than diatoms through diatom-adapted, yeast-derived centromeric sequences (CEN/ARS) is not possible yet. Rather, episomal maintenance is a function of species-specific adaptation of yeast centromeric regions that should be optimized before the wider application of episomal vectors in microalgal bioengineering.

## Case Studies for Genetic Engineering in Microalgae

The previous sections reviewed key resources that can augment the bioengineering in microalgae. This section describes the various algal bioengineering research such as: the enhancement of (1) photosynthesis and biomass production, (2) lipid production, (3) the production of biomolecules, and other value-added products.

### Photosynthetic Efficiency and Biomass Production

Enhanced CO_2_ fixation through augmentation of photosynthetic efficiency is the key process to improve microalgae biomass production, a pre-requisite to develop microalgae as the next-generation feed-stock. The carbon fixation is dependent on multiple factors, where selectivity and velocity of RuBisCo enzyme remains one of the major factors. RuBisCo is capable to fix CO_2_ as well as O_2_ into 3-phosphoglycerate and 2-phosphoglycolate, where 2-phosphoglycolate is undesirable and toxic to the cells. The phenomenon called photorespiration occurs in the mitochondrion and peroxisome, which uses 2-phosphoglycolate to release CO_2_. These futile side reactions ultimately hamper the photosynthetic activity. Therefore, attempts have been made to simultaneously improve the selectivity and catalytic rate of RuBisCO through genetic engineering, though with limited success (Du et al., 2003; Spreitzer et al., 2005). Alternatively, the problem of RuBisCO selectivity can be mitigated by controlling the design consideration of cultivation system to enrich the CO_2_ supply. Nevertheless, in order to improve the catalytic rate of RuBisCO, its genetic modification is preferable mode than to select efficient RuBisCO from the diverse pool of natural variants. In one such effort, the small subunit of RuBisCO enzyme of *Chlamydomonas* has been replaced with that of Arabidopsis, spinach, and sunflower to enhance the carboxylation catalytic efficiency and CO_2_/O_2_ specificity (Genkov et al., 2010). Although the hybrid RuBisCO enzyme had 3–11% increase in specificity, the velocity of the enzyme remained same. Likewise, several amino-acid residues have been identified in the conserved region of small subunit of the RuBisCO that can be the potential target for engineering RuBisCo to improve its catalytic efficiency (Du et al., 2000; Spreitzer et al., 2001; Genkov et al., 2006; Genkov and Spreitzer, 2009). In another approach through regulation of RuBisCo activity, the photosynthetic biomass production in *N. oceanica* was substantially enhanced upon overexpression of RuBisCO activase (Wei et al., 2017a). Beside RuBisCO the other relatively low abundant enzymes of Calvin cycle regeneration phase, such as fructose−1,6-bisphosphatase (FBPase), fructose 1,6-bisphosphate aldolase (FBA), and sedoheptulose 1,7-bisphosphatase (SBPase) are the prime target to manipulate the photosynthetic activity. Recently the engineering of Calvin cycle through the overexpression of cyanobacterial FBA was found to enhance the photosynthetic capacity of *C. vulgaris* (Yang et al., 2017). Similarly, the overexpression of *Chlamydomonas* SBPase was reported to improve the photosynthetic activity in *Dunaliella bardawil* (Fang et al., 2012). The FBPase was found to enhance the photosynthetic efficiency upon overexpression in higher plants (Tamoi et al., 2006). However, its overexpression in *Chlamydomonas* had detrimental effect on growth and photosynthetic activity under high CO_2_ photoautotrophic conditions. This was mainly due to the reduced amount of glyceraldehyde−3-phaspahte because there was enhanced conversion of fructose−1,6-bisphosphate into fructose−6- phosphate (Dejtisakdi and Miller, 2016). This indicates that in microalgae the reaction catalyzed by the FBPase is not a rate limiting one that can be targeted to improve the photosynthetic efficiency and concomitant biomass accumulation.

The photosynthetically efficient microorganisms operate the CO_2_-concentrating mechanisms (CCMs) to increase the CO_2_ concentration in the proximity of RuBisCO, which eventually reduce the photorespiration and promote carboxylation. In comparison to the terrestrial plants, the green microalgae have efficient CCM because of sequestration of the enzymes of photosynthetic machinery in the pyrenoid or peroxisome (Mackinder, 2018; Hennacy and Jonikas, 2020). Several functional and regulatory factors have been identified, which are responsible to facilitate the carboxylation reaction of RuBisCO through CCM. Among these factors *CIA5*, transporter of inorganic carbon (Ci) and carbonic anhydrases (CA) are considered as the targets for manipulation to increase the photosynthetic performance and eventually biomass yield (Moroney et al., 2011; Wang et al., 2015; Yamano et al., 2015; Gee and Niyogi, 2017). However, there are no such reports on successful engineering of CCM components in microalgae, and thus it remains a challenge to enhance the carbon fixation process.

**Figure 2:**
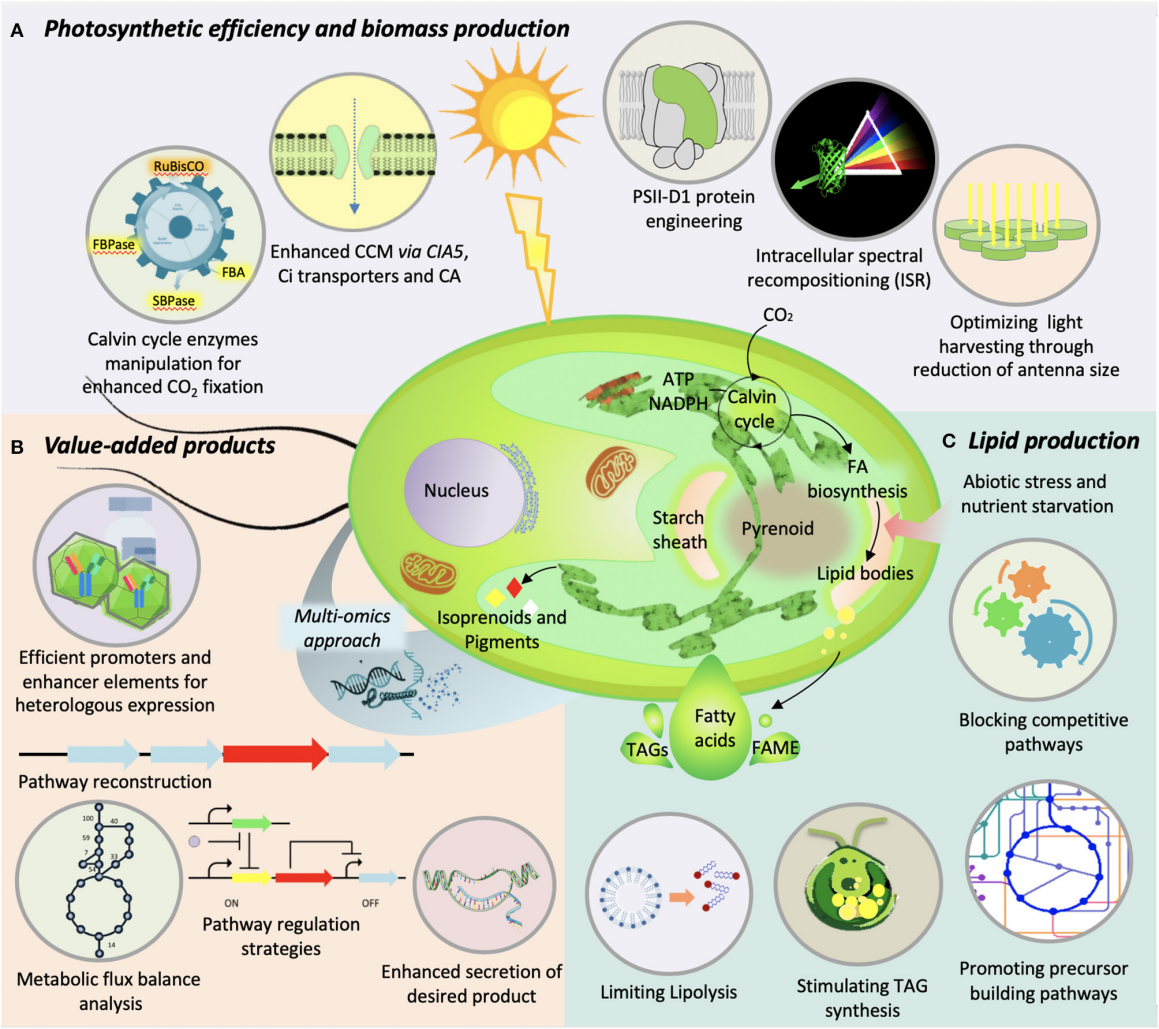
Illustration of various genetic-engineering strategies used in microalgae to improve **(A)** photosynthetic efficiency and biomass production, **(B)** value-added product synthesis, and **(C)** lipid production.

On the other hand, the cultivation of microalgae at high cell density often encounters a problem of photo-limitation because of light shading. The high light intensity at the surface cell layers saturates the photosynthetic process and causes photoinhibition, whereas excess energy is dissipated through non-photochemical quenching. Meanwhile, the low-light intensity at the lower layer of cells compels them to perform photorespiration instead of photosynthesis. This uneven distribution of the light intensity results in suboptimum photosynthetic efficiency that eventually reduces the biomass yield. Reducing the size of antenna or light-harvesting complex is one of the approaches that has the potential to improve the light transmission and light absorption capacity. For instance, the reduction of chlorophyll *b* content and consequent reduction of antenna size in *Chlamydomonas* through RNAi-mediated silencing of *chlorophyllide a oxygenase*, resulted in enhanced photosynthetic activity and higher growth rate as compared to chlorophyll *b* mutant under saturating light conditions (Perrine et al., 2012). Similarly, the *C. vulgaris* mutant with truncated antenna size and reduced chlorophyll *a* and *b* content, generated through random mutagenesis of chloroplast signal recognition particle (*CpSRP43*), exhibit enhanced photosynthetic efficiency associated with reduced non-photochemical quenching and higher biomass yield (Shin et al., 2016b, 2017). The engineering of photosystem II protein D1 isoform in *Chlamydomonas* showed enhanced photosynthetic efficiency under saturating light conditions (Vinyard et al., 2014). In another novel approach, the diatom *P. tricornutum* was engineered to establish a concept of intracellular spectral recompositioning for improved light absorption and consequent higher biomass production (Fu et al., 2017). In this case, the overexpressed green fluorescent protein absorbs the excess blue light energy from incident light and subsequently emits energy as green light that can be harvested by accessory pigments. Thus, spectral recompositioning eventually improves the light absorption and reduces the non-photochemical quenching and may mitigate the problem of photoinhibition at high cell density cultures through deeper penetration of emitted green light. A similar ecological mechanism has been observed in the coral-algae symbionts to acclimatize deep water light environment by facilitating homogenous distribution of available light energy (Smith et al., 2017). Although significant information is availed through genetic engineering to get insight into the photosynthetic efficiency, most of these leads are from the model algal systems. Moreover, this information is yet to be applied for large scale applications. In addition, the design consideration of cultivation system has significant effect on the photosynthetic efficiency and eventually on productivity. Therefore, there is a need of more comprehensive and cumulative approach, such as fine tuning the flux balance of Calvin cycle toward enhanced CO_2_ fixation or perturbation of multiple targets at once to get a synergistic effect. The various strategies to improve photosynthetic efficiency and biomass production are illustrated in Figure 2.

### Lipid Production

Lipids from microalgae are at the center of attention due to their yield and nutraceutical importance. The quantity, quality, and the type of lipids synthesized by microalgae not only help in diversifying their application but influence the biodiesel properties if chosen for the fuel purpose (Shekh et al., 2016). For researchers working in this area, lipid productivity remains a key parameter for strain selection. In fact, the kind of lipids a microalga accumulates plays a key role in its commercial utilization for food, feed, or fuel purpose. Over the years, a trade-off between enhancing microalgal lipid content by various means without compromising the lipid productivity was targeted. Various augmentations in environmental, nutritional, and physiological conditions for cultivation of microalgae, as well as genetic manipulations, have been attempted for enhanced lipid production (Figure 2). However, genetic engineering of the robust strains for enhanced lipid production remains one of the most viable options to improve the process economics. In the recent past, various genes involved in lipid biosynthesis were knocked-out or overexpressed to examine their effects on lipid accumulation. *Acetyl-CoA Carboxylase* (*ACCase*), which encodes enzyme for fatty acid synthesis, was overexpressed for the first time in 1996 by Dunahay et al. (1996). Even though the overexpression of ACCase was characterized by 2- to 3-fold increase in ACCase activity, it could not lead to increased lipid accumulation (Sheehan et al., 1998). However, upregulation of ACCase in tandem with malic enzyme, which catalyzes malate to pyruvate conversion, was effective in enhanced lipid accumulation in *D. salina* (Talebi et al., 2014). Overexpression of diacyl glycerol acyl transferase, which catalyzes the final step in TAG synthesis, is often-used strategy, which also resulted in lipid enhancement (Niu et al., 2013; Iwai et al., 2014; Li et al., 2016a). Also, the enhanced expression of pyruvate dehydrogenase, acetyl-CoA synthase, phosphoenolpyruvate carboxylase, NAD(H) kinase, and glycerol kinase has resulted in hyperaccumulation of lipids in various microalgal species. Simultaneous expression of multiple acyl transferases from *S. cerevisiae* and *Yarrowia lipolytica* in *Chlorella minutissima* resulted in twofold lipid accumulation (Hsieh et al., 2012). Overexpression of RuBisCO activase in *N. oceanica* has resulted in an increase in the productivity, thereby increasing lipid accumulation (Wei et al., 2017a). Inhibiting the expression of a multifunctional lipase/phospholipase/acyltransferase in *T. pseudonana* resulted in enhanced lipid accumulation without compromising the growth (Trentacoste et al., 2013). On the other hand, it is known that the transcriptional regulation can influence metabolomic flux of the system as transcription factors can target multiple regulatory points in a metabolic pathway. Overexpression/knockdown of transcription factors targeting the upregulation of lipid biosynthesis genes may accumulate higher lipids. In one of the efforts, knockdown of a single transcription regulator *ZnCys* in *N. gaditana* resulted in twofold increase in lipid content (Ajjawi et al., 2017). Strategies to prevent the degradation of synthesized lipids were also studied to improve lipid yields. A knock-out mutant of the phospholipase A2 gene (*C. reinhardtii*) had the total lipid content increased up to 64.25% (Shin et al., 2019). In another study, a 10-fold increase in TAG was reported upon silencing the *cht7* gene encoding a TAG lipase (Tsai et al., 2014). Most recently, CRISPR/Cas9-based technology for gene manipulation in *C. vulgaris* was used wherein a fragment of Cas9 with sgRNA designed on omega-3 fatty acid desaturase (*fad3*) gene was constructed. This has resulted in 46% (w/w) higher accumulation of lipid content (Lin and Ng, 2020). Even though various studies to genetically engineer microalgae for enhanced lipid accumulation have been attempted, they are mostly restricted to model and/or selected microalgae strains. The recent advancements in gene-editing technologies especially CRISPR/Cas9 may allow the gene manipulations in commercially important oleaginous strains so as to improve the process economics.

### Biomolecules and Value-Added Products

Beside lipids, microalgae are rich in biomolecules such as carotenoids with potential application in human health. The accumulation of biopigments in microalgae is known to be affected by various biotic and abiotic factors, the details of which have been recently reviewed by Saini et al. (2020). Since the carotenoid biosynthesis pathway has been extensively studied, the metabolic engineering, in addition to the mutant screening, has been applied to enhance the production of carotenoids in microalgae. Perturbing the pathway enzymes such as phytoene synthase and phytoene desaturase, microalgae have known to enhance the production of carotenoids (Steinbrenner and Sandmann, 2006; Couso et al., 2011; Dambek et al., 2012; Tran et al., 2012; Liu et al., 2014; Eilers et al., 2016; Galarza et al., 2018). In addition, several other enzymes involved in the subsequent steps of the carotenoid pathways have also been targeted. For instance, the overexpression of *Haematococus pluvialis* gene encoding β-carotene ketolase in *Dunaliella salina* resulted in production of astaxanthin (Anila et al., 2016). The downregulation of squalene epoxidase through RNAi in *Chlamydomonas* was found to accumulate squalene (Kajikawa et al., 2015). Similarly, the knock-out mutant of *zeaxanthin epoxidase* in *Chlamydomonas* had significantly higher zeaxanthin content than the wild type (Baek et al., 2018). However, diverting the flux toward desired metabolites is not that simple and may require perturbation of multiple genes of a pathway. In one such recent example, the overexpression of three exogenous enzymes, namely oxidosqualene cyclase (from *Lotus japonicus*) and cytochrome P450 along with its native reductase (from *Medicago truncatula*) in *P. tricornutum*, leads to the production of triterpenoids *viz*. lupeol and botulin (D'Adamo et al., 2019). Similarly, the production of sesquiterpenoids and diterpenoids through genetic engineering of *Chlamydomonas* has also been reported (Lauersen et al., 2016, 2018; Wichmann et al., 2018). The introduction of additional copy of gene encoding gateway enzyme of terpenoid pathway, 1-deoxy-D-xylulose 5-phosphate synthase (*dxs*), resulted in enhanced accumulation of fucoxanthin in *P. tricornutum* (Eilers et al., 2016). However, the hyperaccumulation of carotenoids or any other secondary metabolites sometimes causes feedback inhibition. Therefore, generating an additional metabolic sink (in a place other than the site of production) or expressing the flux controlling enzyme(s) that can resist the feedback inhibition could be the possible strategies. However, this strategy may get limited by the lack of information on transporters or the flux controlling enzymes. Here the genomic information can substantially improve the scenario of metabolic engineering in microalgae.

The algal nuclear or chloroplast engineering has been extensively carried out using synthetic biology approach for the production of recombinant proteins having therapeutic properties. Some of the inherent features of algae such as lack of infectious agents or toxins, efficient folding of complex proteins and scope for the development of whole algae as low-cost oral vaccine, makes them ideal platform for heterologous production and offer several advantages over the better established microbial and mammalian systems. Although, most of the chloroplast transformation attempts have been made in the model microalgae *Chlamydomonas*, the successful chloroplast engineering has also been demonstrated in few other microalgal species [reviewed by Siddiqui et al. (2020)]. It was reported that over 100 different recombinant proteins have been successfully expressed in algal chloroplast. Among these recombinant proteins, the vaccines, antibodies and immunotoxins, and therapeutic proteins are the major targets (Dyo and Purton, 2018). The production of whole algal cells as oral vaccines specially for farm animals, where the fusion of protein adjuvant (cholera toxin B subunit: CTB) to the N-terminus of the antigen facilitates the antigen absorption through gut epithelium, provided an alternative low-cost vaccination strategy. Moreover, this bioencapsulation of therapeutic proteins has advantages of long-term storage at room temperature and also protects them from the degradation in animal stomach (Dreesen et al., 2010; Gregory et al., 2013). Besides all these advantages, the yield of the recombinant proteins is still a major concern to adopt algae as protein production platform. Although several recombinant proteins have been successfully produced through genetic engineering of nuclear genome, a much lower success rate with production yield of only up to 0.25% of total soluble proteins was reported (Scranton et al., 2016). In comparison, the production of proteins through chloroplast engineering may reach up to 0.1–5% of total soluble protein (Dyo and Purton, 2018). Nevertheless, the nuclear expression of the protein offers some interesting features such as ability to target the protein to secretory pathway or to the specific organelles that may also allow the post-translational modification of the proteins (Lauersen et al., 2015). The various signal peptides have been used to target the proteins either to secretory pathway or to an organelle. Recently, the performance of two *in-silico* identified signal peptides (1,3-α-glucosidase and SAD1p derived) to efficiently secrete expressed reporter protein in *C. reinhardtii* has been successfully demonstrated (Molino et al., 2018). The different promoters and their respective 5' UTRs as well as their synthetic variants have been used to derive the expression of transgene in order to mitigate the constrains of inefficient transgene expression in microalgae (Coragliotti et al., 2011; Specht and Mayfield, 2013; Gimpel et al., 2015). For example, the use of strong promoter such as 16S ribosomal RNA fused to 5'UTRs of endogenous photosynthetic genes can be used to enhance the expression of transgene to some extent (Rasala et al., 2011). However, the performance of the endogenous 5′UTRs to translate the gene of interest is still the major constrain. The intrinsic features of photosynthetic genes derived 5′UTRs are also responsible for feedback regulation of translation. It does so through “control by epistasis of synthesis” that prevent overaccumulation of protein subunit in the absence of other subunits of the protein assembly (Coragliotti et al., 2011). In addition, the constitutive expression of the transgene negatively impacts the growth of the transgenic algae as an extra metabolic burden. Therefore, the use of inducible promoter to tightly regulate the expression of transgene could be the better strategy to improve the growth efficiency, and hence the productivity of desired product (Fajardo et al., 2020). The various promoters used so far in the microalgal research are given in Table 3. The advancement in the synthetic biology and our understanding on the regulation of protein synthesis in microalgae will enable us to improve the protein expression level in microalgae so as to make microalgae a feasible host system for commercial application. The various strategies to improve production of bioactive of interest in microalgae are illustrated in Figure 2.

**Table 3:** List of endogenous and heterologous promoters used in microalgae research.

**Target species**	**Promoters**	**Nuclear (*N*)/ chloroplast (*C*) expression**	**Salient features**	**References**
*Ankistrodesmus convolutus*	AcRbcS promoter	*N*	Light-regulated promoter	Thanh et al., 2012
*C. reinhardtii*	ARG7 promoter	*N*	Strong promoter	Specht et al., 2015
	β-TUB2 promoter	*N*	Constitutive promoter	Crozet et al., 2018
	CABII-1	*N*	Light-dependent promoter	Doron et al., 2016
	Cyc6 and Cpx1 promoter	*N*	Copper- and oxygen-dependent promoter	Quinn et al., 2000
	CrGPDH3 promoter	*N*	Salt inducible promoter	Beltran-Aguilar et al., 2019
	Fea1 promoter	*N*	Iron-responsive promoter	Barjona do Nascimento Coutinho et al., 2019
	HSP70A-RBCS2 promoter	*N*	Strong hybrid promoter	Lauersen et al., 2015
	HSP70A promoter	*N*	Strong promoter activity	Schroda et al., 2000
	psaD promoter	*N*	Light-responsive constitutive promoter	Crozet et al., 2018
	sap11 promoter	*N*	Synthetic strong promoter	Scranton et al., 2016
	RBCS2 promoter	*N*	Strong promoter activity	Lumbreras et al., 1998
	psaA promoter	*C*	Light-responsive strong promoter	Michelet et al., 2011
	psbA promoter	*C*	Light-responsive strong promoter	Rasala et al., 2011
	psbD promoter	*C*	Light-responsive strong promoter	Rasala et al., 2011
	atpA promoter	*C*	Constitutive promoter activity	Rasala et al., 2011
	16S promoter-psbA 5' UTR	*C*	Strong promoter	Rasala et al., 2011
	rbcL promoter	*C*	Light-responsive strong constitutive promoter	Rasala et al., 2011
*Chaetoceros gracilis*	Lhcr5 promoter	*N*	Constitutive promoter	Ifuku et al., 2015
*C. vulgaris*	CaMV35S promoter	*N*	Constitutive promoter	Chow and Tung, 1999
	CvpsaD promoter	*N*	Light-responsive promoter	Kim et al., 2018
*Chlorella ellipsoida*	Ubi1- **Ω** promoter	*N*	Strong constitutive expression	Chen et al., 2001
*Cyclotella cryptica*	ACCase promoter	*N*	Constitutive promoter	Dunahay et al., 1996
*Cylindrotheca fusiformis*	fruα3 promoter	*N*	Strong constitutive expression	Fischer et al., 1999
*D. salina*	LIP promoter	*N*	Light-inducible promoter	Baek et al., 2016b
	GAPDH promoter	*N*	Constitutive promoter	Doron et al., 2016
*Fistulifera sp*.	fcpB promoter	*N*	Constitutive promoter	Muto et al., 2013
	H4 promoter	*N*	Constitutive promoter	Muto et al., 2013
*H. pluvialis*	CaMV 35S	*N*	Constitutive promoter	Kathiresan et al., 2009
	Ptub promoter	*N*	Strong promoter	Yuan et al., 2019
	rbcL promoter	*C*	Light-responsive strong constitutive promoter	Gutiérrez et al., 2012
*P. tricornutum*	CaMV 35S promoter	*N*	Constitutive promoter	Chow and Tung, 1999
	U6 promoter	*N*	Constitutive promoter	Serif et al., 2018; Stukenberg et al., 2018
	Lhcf promoter	*N*	Light-dependent promoter	Lepetit et al., 2010
	NIT promoter	*N*	Ammonium inducible promoter	Chu et al., 2016
	pPhAP1 promoter	*N*	Strong promoter	Lin et al., 2017
	Pt211 promoter	*N*	Strong constitutive promoter	Zou et al., 2018
	fcp promoter	*N*	Constitutive promoter	Watanabe et al., 2018
	V-ATPase promoter	*N*	Strong constitutive promoter	Watanabe et al., 2018
	ef2 promoter	*N*	Constitutive promoter	Seo et al., 2015
	HASP1 promoter	*N*	Strong constitutive promoter	Erdene-Ochir et al., 2019
	rbcL promoter	*C*	Light-responsive strong constitutive promoter	Xie et al., 2014
*N. oceanica*	β-tubulin promoter	*N*	Constitutive promoter	Li et al., 2014a
	CMV viral promoter	*N*	Constitutive promoter	Osorio et al., 2019
	ef promoter	*N*	Constitutive promoter	Poliner et al., 2018
	Ribi promoter	*N*	Bidirectional strong constitutive promoter	Poliner et al., 2018
	EM7 promoter	*N*	Constitutive promoter	Osorio et al., 2019
	NIT promoter	*N*	Ammonium inducible promoter	Jackson et al., 2019
	VCP promoter	*N*	Constitutive promoter	Li et al., 2014a
	rbcL promoter	*C*	Light-responsive strong constitutive promoter	Gan et al., 2018
*N. gaditana*	TCT promoter	*N*	Constitutive promoter	Ajjawi et al., 2017
	RPL24 promoter	*N*	Constitutive promoter	Ajjawi et al., 2017
	4ALL promoter	*N*	Constitutive promoter	Ajjawi et al., 2017
	EIF3 promoter	*N*	Constitutive promoter	Ajjawi et al., 2017
*N. oculata*	HSP70A-RBCS2 promoter	*N*	Strong hybrid promoter	Shih et al., 2015
*N. salina*	TUB promoter	*N*	Constitutive promoter	Koh et al., 2019
	UEP promoter	*N*	Constitutive promoter	Koh et al., 2019
*T. pseudonana*	Lcfs9 promoter	*N*	Constitutive promoter	Poulsen et al., 2006
	NIT promoter	*N*	Nitrate inducible promoter	Poulsen et al., 2006
*Volvox carteri*	LHCBM1 promoter	*N*	Constitutive promoter	Tian et al., 2018
	nitA promoter	*N*	Nitrate inducible promoter	von der Heyde et al., 2015
	ISG promoter	*N*	Developmental stage (embryonic inversion) specific promoter	Hallmann and Sumper, 1994
	Arylsulfate promoter	*N*	Sulfur starvation inducible promoter	Hallmann and Sumper, 1994

## Risk Assessment, Biosafety, and Regulatory Issues

Though genetic engineering is considered as one of the most potent tools to augment production of commercially valuable metabolites in microalgae, it inevitably invites varying opinions on the safe use of genetically modified (GM) algae for consumption and environment. On the contrary, several algal performance-improvement strategies, which could have environmental and ecological threats, are in use without much debate. In many parts of the world, strict laws/policies require transgenic/recombinant algae to undergo regulatory compliances. When research and policy complement each other, technological advances move at a rapid pace. In this case, even if various researchers across the globe are working on strain improvement for enhanced microalgae performance through genetic modifications, their commercial use is restricted. Reports indicate that the Florida-based biotechnology company named Algenol was given approval for use of GM cyanobacteria for cultivation in outdoor closed-photobioreactor. At the same time, the secretariat of the Convention on Biological Diversity in its 2015 report has raised the concerns over strict physical containment of these GM microorganisms by the company (https://www.cbd.int/ts/cbd-ts-82-en.pdf). It is arguably said that the U.S. Environmental Protection Agency (US-EPA) relies upon a regulatory regime-Toxic Substances Control Act (TSCA), which has become outdated and is incapable of assessing the novel risks arising out of the new biotechnological inventions. Under TSCA, companies are only required to file a Microbial Commercial Activity Notice for commercialization of a new GM microorganism. Till date, no outdoor cultivation of GM microalgae is reported probably due to various predictable and unexpected risks associated with its open cultivation (Nethravathy et al., 2019). Cultivation of GM microalgae possesses several risks, which includes spills that may become uncontrollable. These algae upon proliferation compete with natural species and may outgrow them. In fact, the genetically modified traits of the organisms may provide them the competitive advantage in natural ecosystem. Risks also exist for genetic contamination /interbreeding with wild-type or sexually compatible strains. Threats of harmful algal blooms, negative impacts on ecosystem, increased selection pressure, horizontal gene transfer, health and environmental impacts, unpredictable future of GM traits, loss of management control, and ethical concerns are some of the major concerns associated with cultivation of GM algae (Nethravathy et al., 2019). Apart from regulations for the use of GM algae, strict biosecurity laws are required to safeguard the importation of foreign species (GM and/or wild-type) to the local environment. Though the import and use of foreign algae strains, which are non-native to local environment, have a very little regulatory control, the associated risk of these strains dominating the local species must be seriously considered (Campbell, 2011). The concrete environmental risk due to algal spills must not only be limited to the GM aspect of the strains. Further, assessment needs to be carried out considering fitness of invading foreign species in comparison with local algal community along with intricacies and population stability characteristics of the ecological system in question (Henley et al., 2013). To further improve the situations for the use of GM algae, in-depth cost-benefit analysis of GM microalgae to society and environment must be carried out. Strict monitoring of the handling and cultivation process with health and environmental risk assessment analysis are integral to design the biosafety regulations for GM microalgae. Since GM algae are considered as one of the solutions to overcome techno-economic challenges in algal industry, it is imperative that various stakeholders including business promoters and policy makers collectively reach to a consensus on a road map for the use of GM algae in future. Various federal governments across the globe must bring in place the policies and regulations that govern the safe use of GM algae for human and environmental benefit.

## Conclusion and Future Prospects

Currently, economically feasible, environmentally sustainable, and replicable microalgal processes with higher technology readiness levels are required for ease of doing algal business. To improve the economic feasibility of the algal processes, the genetic engineering of microalgae is at forefront for development of robust microalgal strains. Advances in the high-throughput technologies and molecular biology tools have facilitated the biotechnological approach to engineer the microalgal strains for performance improvement. The synergy of microalgal multi-omics datasets and the advanced molecular tools offer a rapid and predictable strategic path for the strain improvement. In this review, various microalgal resources such as genome sequence, mutant libraries, high-throughput screening methodologies, and genetic tools and techniques were summarized that holds the potential for the development of microalgae as a next-generation renewable resource. In addition, the catalog of various *omics* study under different conditions across the diverse microalgal species is generated (Table 1). Despite the variation in the inter- and intraspecies *omics* datasets, the several conserved factors can be mined to predict the biological outcomes with the comprehensive use of system biology approach. Various omics-based approaches must aim to enhance microalgal capacities to produce high value metabolites. Future research may focus on developing purpose-specific robust bioengineered strains for high photosynthetic efficiency, high CO_2_ fixation, and high biomass productivities. Also, targeted enhancement of low-volume, high-value metabolites of biomedical applications from microalgae must be considered using genetic engineering.

Though the genetic engineering of microalgae holds great potential to improve process economics, it is limited mainly due to the unavailability of the genetic information for robust and commercially suitable strains. In recent times, rapid advances in DNA synthesis, genetic manipulation tools and techniques, availability of functional genomes have improved the chances to better engineer microalgae with complex functions. However, the lack of genetic strain design principles is still hurting the progress in this area. Further, once the genetically improved strains are developed, safety to human health and environment will define its commercial success. Therefore, it is recommended that strict regulations and monitoring should be in place to evaluate the environmental and human health risk of using GM microalgae particularly in outdoor cultivation. Here, the recent development in precise genome editing technologies such as non-transgenic and marker-free CRISPR has the potential to revolutionize the microalgal bioengineering for the production of non-GMO algal products. The non-GMO tag to the bioengineered microalgae is expected to improve the biosafety and alleviate the regulatory issues associated with the usage of GM microalgae. In view of uncertainty within the academic and industrial community regarding the regulations for the use of GM strains, and the inadequacy of current regulations for the use of GM algae, a clear road map for regulatory regime covering the commercial use of GM microalgae is urgently required. Since the robustness of non-model microalgae species has advantages in commercial and industrial applications over model species, there is a need to develop advanced research tools for the non-model microalgal species. Moreover, to improve the economic competitiveness of algal-derived products, the development of efficient extraction methods or the use of whole cells is needed. Indeed, beside all the developments, bio-prospection for novel and robust microalgae with industrial viability must continue.

## Author Contributions

GK: conceptualization, writing—original draft preparation, writing—reviewing, editing, and supervision. AS: conceptualization, writing—original draft preparation, writing—reviewing, and editing. SJ: investigation—data collection and writing—original draft preparation. YS: visualization and writing—original draft preparation. RK: writing—original draft preparation. TS: writing—reviewing, editing, and supervision. All authors contributed to the article and approved the submitted version.

## Conflict of Interest

The authors declare that the research was conducted in the absence of any commercial or financial relationships that could be construed as a potential conflict of interest.
